# Dental Implants in Medically Compromised Patients Undergoing or After Receiving Anti‐Resorptive or Radiotherapy: Retrospective Clinical and Radiographic Data

**DOI:** 10.1111/clr.70040

**Published:** 2025-09-03

**Authors:** Christian Mertens, Fabian Staudinger, Maximilian Smielowski, Thomas Rückschloss, Gregor Schnug, Jürgen Hoffmann, Oliver Ristow

**Affiliations:** ^1^ Department of Oral‐ and Cranio‐Maxillofacial Surgery Heidelberg University Hospital Heidelberg Germany

**Keywords:** dental implants, diphosphonates, follow‐up studies, radiotherapy, risk assessment, risk factors, survival rate

## Abstract

**Objective and Aim:**

A retrospective study to evaluate the clinical and radiological outcomes of dental implants placed in compromised patients who have undergone antiresorptive therapy (AR) or head and neck radiotherapy (IR).

**Material and Methods:**

Dental implant placement was evaluated in compromised patients undergoing or after receiving AR/IR therapy following specific preventive measures: antibiotic prophylaxis (2 days before and 5 days after surgery) and primary wound closure with submerged healing for 4 months. The primary outcome was implant survival during the observation period. Secondary outcomes included marginal bone loss, occurrence of osteonecrosis, and factors influencing implant survival.

**Results:**

A total of 92 patients (59 IR, 32 AR) with 369 dental implants were included in the study. During a mean follow‐up period of 25 months (SD: 16), 23 implants were lost (IR: 21, AR: 2). Implant survival rates were 92% and 98% for IR and AR, respectively. Identified risk factors for implant failure included placement in neomandibular/−maxillary sites, maxillary implantation, implant diameter < 4.2 mm, active smoking, and diabetes mellitus. The mean marginal bone loss was 0.57 mm (SD: 1.51) in the IR group and −0.09 mm (SD: 1.17) in the AR group.

**Conclusion:**

With comprehensive risk assessment and careful evaluation of implant indications, dental implants can serve as an effective dental rehabilitation option for patients undergoing or after receiving AR/IR therapy, provided strict adherence to preventive measures is maintained. Implants placed following AR therapy demonstrate higher survival rates and reduced marginal bone loss compared to those placed after IR therapy.

## 1 Introduction

Dental implants have become essential for the rehabilitation of edentulous and partially edentulous patients, offering predictable outcomes and high survival rates under optimal conditions. The success of implant therapy is primarily influenced by the quantity and quality of the supporting bone, which is crucial for long‐term stability and function. In recent years, however, implantology has expanded to include patients with compromised bone conditions. Although implant survival rates in these populations may be lower, significant gains in oral function, aesthetics, and overall quality of life can still be achieved (Li and Leung 2024).

Patients undergoing or having completed antiresorptive (AR) or irradiation (IR) therapy represent a distinct subgroup with compromised bone health. According to the infection‐dependent pathogenesis theory, preventing bone infections, which are often initiated by dental inflammation, periodontitis, or even minor trauma from prosthesis pressure points, is paramount for these patients (Otto et al. 2021). Consequently, preemptive removal of non‐restorable teeth is recommended before initiating AR/IR therapy, (Ristow et al. 2021) meaning that many patients already present with a severely compromised dental status at the outset (Ristow, Ruckschloss, et al. 2023).

Post‐therapy complications further obscure the situation. Late effects of radiation, such as tooth loss from radiation caries, and the need for continuous management of non‐preservable dental foci during and after AR/IR therapy contribute to progressive tooth loss. These factors underscore the critical role of prosthetic rehabilitation in this patient group (Schiegnitz et al. 2018).

Rehabilitating these patients requires a careful balance. On the one hand, mucosa‐supported prostheses help preserve the integrity of both the mucosa and fragile bone; on the other hand, they may still pose a risk for osteonecrosis of the jaw (ONJ) due to localized pressure points. Implant‐supported prostheses, while potentially reducing the risk of ONJ by eliminating such pressure, involve surgical procedures within a compromised bone environment and carry their own risks, particularly peri‐implant infections (Schiegnitz et al. 2018), and the uncertainty of achieving reliable osseointegration in metabolically impaired bone (Nisi et al. 2024).

Given that implant placement in these high‐risk populations is elective, a rigorous assessment of the indication, meticulous treatment planning, and adherence to evidence‐based protocols are essential to minimize complications and optimize outcomes (Nisi et al. 2024; Park et al. 2024).

The objective of this retrospective study was to evaluate the clinical and radiological outcomes of dental implants placed in compromised bone in patients who have undergone AR/IR treatment, following specific preventive measures including antibiotic prophylaxis and primary wound closure with submerged healing. The focus was on key metrics, including implant survival rates, marginal bone loss, and the incidence of ONJ and peri‐implantitis, to provide insights into the viability and safety of implant therapy in these challenging scenarios.

## 2 Patients and Methods

The aim of this retrospective study was to evaluate the clinical and radiological outcomes of dental implants placed between 2018 and 2023 at the Department of Oral and Craniomaxillofacial Surgery of Heidelberg University Hospital in compromised bone of patients who had undergone AR therapy or head and neck IR. The study protocol was approved by the Ethics Committee of the Medical Faculty (approval no. S‐373/2022) and conformed to recognized ethical standards, including the Declaration of Helsinki. Data are reported in accordance with STROBE guidelines.

Inclusion criteria were:

- Patients under/after AR therapy (e.g., bisphosphonates or Denosumab) or patients with a history of head–neck IR.
- Patients who received dental implants following a standardized protocol with the necessary preventive measures.
- Patients who completed prosthetic rehabilitation and attended the scheduled follow‐up appointment.

Exclusion criteria were:

- Patients who received IR or AR therapy after implant insertion.
- Patients who did not complete prosthetic rehabilitation.
- Patients who did not attend follow‐up appointments.
- Patients who underwent simultaneous bone or soft‐tissue augmentation procedures.
- Patients with the manifest diagnosis of ONJ.

Outcome parameters:

The primary outcome was implant survival throughout the entire observation period.

Secondary outcomes were:

Peri‐implant marginal bone loss:

Bone levels at the mesial and distal aspects of all implants were measured at the following time points:

- *T*_0_: within 1 month after implantation
- *T*_1_: 6 months after implantation
- *T*_2_: 12 months after implantation
- *T*_max_: maximum duration of the observation period

The differences in bone levels between *T*_0_ and *T*_1_, *T*_2_, and *T*_max_ were calculated for each implant to assess bone resorption over time.

Biological complications:

The development of ONJ or peri‐implantitis was evaluated by assessing clinical parameters at 8 weeks, 6 months, and 1 year post‐implantation. The following parameters were recorded:

- Dehiscence (yes/no)
- Infection (yes/no)
- Bleeding on probing (BOP) (yes/no)
- Probing depths (categorized as < 3.5 mm or > 3.5 mm)

### 2.1 Surgical Approach

All patients underwent a standardized presurgical examination and surgical procedure, identical for both study groups. Prior to surgery, patients underwent clinical and radiographical evaluations to identify any conditions requiring pre‐surgical treatment, such as periodontal disease or apical lesions. Patients were also assessed for signs of ONJ and to evaluate their oral hygiene status.

To determine the available bone volume for implant placement and to exclude osteonecrosis, cone‐beam computed tomography (CBCT) was performed prior to surgery.

Surgical interventions were performed following our in‐house protocol, which was adapted in accordance with the German guidelines under strict adherence to preventive measures (Groetz et al. 2016; Ristow et al. 2021; Schiegnitz et al. 2018). Antibiotic prophylaxis was initiated 2 days preoperatively and continued for 5 days postoperatively. Patients without penicillin allergies received amoxicillin‐clavulanic acid (875/125 mg) twice daily, while those with penicillin allergies were prescribed clindamycin (600 mg) three times daily. In addition, Chlorhexidine irrigation therapy was administered 2 days before surgery and continued until suture removal (10–14 days postoperatively).

Under local anesthesia, a crestal incision was made, with vertical releasing incisions added as needed to achieve primary wound closure. Implant sites were prepared according to the manufacturer's protocol, taking care to avoid excessive insertion torque. Only crestal implants (Astra Tech Implant System EV, Dentsply Sirona, York, PA, USA or Straumann BLT, Straumann Holding AG, Basel, Switzerland) were placed at the level of the marginal bone or slightly subcrestally. Each implant was sealed with a cover screw and left for submerged healing. Special attention was given to achieve tension‐free primary wound closure, including periosteal releasing incisions when necessary.

Patients were instructed to follow a soft diet and to avoid wearing their removable provisional prostheses for at least 3 weeks and not until proper adaptation and relining were achieved.

Sutures were removed 10–14 days after surgery, followed by a second follow‐up 1 week later. If the soft tissue was intact, patients were scheduled for second‐stage surgery. In cases of compromised healing, local wound care and additional follow‐ups were arranged.

Second‐stage surgery was performed under local anesthesia after a four‐month healing period. Particular attention was given to ensuring an adequate band of keratinized mucosa around the implants. In some cases, additional soft‐tissue procedures, such as vestibuloplasty with free gingival grafts or split‐thickness skin grafts from the thigh, were required. If soft‐tissue procedures were indicated, they were performed before connection of the final abutment. No soft‐tissue procedures were performed in conjunction with implant insertion.

After prosthetic rehabilitation, patients were monitored regularly, often in conjunction with their monthly oncology follow‐ups to ensure ongoing care.

### 2.2 Study Size

The study size was determined by a comprehensive search of the institutional patient database for individuals with a documented history of AR therapy or head–neck IR who subsequently received dental implants according to the predefined preventive measures. Only patients meeting the strict inclusion criteria for both risk groups were included, ensuring that the sample accurately reflected the targeted population.

### 2.3 Assessment of Variables

Data on patient demographics, implant survival, marginal bone loss, and clinical complications (e.g., dehiscence, infection, bleeding on probing and probing depths) were obtained from electronic health records, standardized clinical charts, and radiographic examinations (panoramic radiographs and CBCT). Marginal bone loss was measured at the mesial and distal aspects of each implant using calibrated digital imaging software following a standardized protocol. Clinical parameters were recorded during routine follow‐up examinations by the same experienced examiner. For both the AR and IR groups, identical methods and criteria were applied to assess each variable, ensuring comparability across groups. This uniform approach to data collection and measurement minimized potential bias and enhanced the reliability of intergroup comparisons.

### 2.4 Statistical Analysis

Statistical analysis was performed using SigmaPlot 12.0 (Systat Software Inc., San Jose, CA, USA) and R‐studio v. 4.3.2 (Posit Software, Boston, MA, USA). To describe the patient cohort, descriptive statistics, including minima, maxima, medians, arithmetic means, and corresponding standard deviations, were calculated.

Kaplan–Meier curves were used to graphically represent implant survival rates. In this study, the event was defined as implant loss. To compare the Kaplan–Meier curves between the AR and IR patient groups, the log‐rank test was applied.

The statistical significance of differences between the various time points was determined using the Mann–Whitney *U* test. The null hypothesis (H_0_) was: “There is no difference between the compared values,” while the alternative hypothesis (H_A_) was: “There is a difference between the compared values.” The smaller the resulting *p*‐value, the more likely the alternative hypothesis (H_A_). Values with *p* < 0.05 were regarded as statistically significant.

For analysis at the implant level, a logistic regression model with regularization (LASSO method) was applied, using 10,000 bootstrap replications. Since the number of independent variables was large, a standard regression model would not perform well due to strong multicollinearity among predictors. To account for the multilevel structure of the data (e.g., multiple implants per patient), the analysis was conducted using a generalized linear mixed model with LASSO regularization (glmmLasso package in R).

The LASSO method incorporates a regularization parameter (*λ*), which helps mitigate the adverse effects of multicollinearity. It is also well‐suited for datasets with many predictors, as it reliably identifies the key risk factors by shrinking non‐relevant coefficients to zero. For the purpose of this analysis, the regularization parameter was fixed at *λ* = 5. This value was chosen as the smallest integer for which the estimation procedure remained numerically stable. The *p*‐values were not computed using a normal approximation but were instead derived using the more precise quantile method, which directly uses the empirical distribution of the bootstrap samples to assess significance. LASSO regularization was applied within each of the 10,000 bootstrap replications, and the coefficients shown in Table 1 represent the means across all replications. As a result, the table does not reflect a single‐step variable selection, which explains why some predictors with smaller or nonsignificant effects are still included in the final output.

**TABLE 1:** Regression coefficients (dependent variable: Implant failure).

Variable	Total cohort		Subgroup	
	Coefficient	*p*	Coefficient	*p*
Intercept	−2.90*	< 0.01	−2.94*	< 0.01
Neomandible implantation	0.51*	< 0.01	0.56*	< 0.01
Implant diameter < 4.2 mm	0.47*	< 0.01	0.52*	< 0.01
Diabetes mellitus	0.45*	< 0.01	0.56*	< 0.01
Current smoker	0.22*	0.02	0.25*	0.02
Maxillary implant	0.22*	< 0.01	0.41*	< 0.01
History of osteonecrosis of the jaw	0.09	0.10	0.01	0.75
Current or former smoker	0.08	0.08	0.05	0.12
Current smoker and alcohol consumption	0.06	0.14	0.06	0.16
Anterior implant position	0.04	0.10	0.02	0.56
Alcohol consumption	0.02	0.42	0.02	0.42
Vestibuloplasty/free gingival graft	0.00	0.86	0.04	0.30
Gender: male	−0.02	0.52	−0.06	0.26
Age > 65	−0.02	0.58	0.06	0.20
Cardiovascular disease	−0.02	0.44	−0.01	0.66
Former smoker	−0.02	0.48	−0.05	0.16
Distal implant position	−0.04	0.10	0.02	0.66
Hyper‐/hypothyroidism	−0.06	0.08	−0.02	0.42
Never smoker	−0.08	0.08	−0.05	0.10
Anticoagulants	−0.16*	0.02	−0.21*	0.02
Hypertension	−0.16*	0.04	−0.16*	0.04
Mandibular implant	−0.20*	0.02	−0.36*	< 0.01
Time interval IR to implantation < 60 months			0.25*	0.02
Time interval IR to implantation < 12 months			−0.28*	< 0.01

*Note:* * *p* < 0.05.

Positive coefficients (*ß*) indicate a positive association between the variable and probability of implant failure. Conversely, negative coefficients suggest a negative (protective) association.

## 3 Results

### 3.1 Demographics

A total of 92 patients were included in this study (39 male, 53 female), with a mean age of 66 years (SD: 11, range 23–87) at implant insertion. Fifty‐nine patients were treated after IR therapy and 32 during/after AR therapy (Table 2).

**TABLE 2:** Patient characteristics and implant survival: Overall and by group.

	Overall	IR	AR
Number of patients (*n*=)	92	59	32
Male (*n*=)	39	35	3
Female (*n*=)	53	24	29
Patient age mean (years)	66	63	72
Patient age min‐max (years)	23–87	23–83	55–87
Number of implants (*n*)	369	265	97
Failed implants (*n*)	23	21	2
Implant survival (%)	94	92	98
Time to failure (month after insertion)	16	15	28

Abbreviations: AR, antiresorptive therapy; IR, irradiation.

The prevalence of comorbidities was as follows: anticoagulant therapy 38% (IR 37%, AR 41%), diabetes mellitus 10% (IR 12%, AR 6%), hypertension 52% (IR 53%, AR 53%) and cardiovascular diseases 43% (IR 39%, AR 56%; Table 3).

**TABLE 3:** Patient characteristics and comorbidities: Overall and by group.

	Overall	IR	AR
Number of patients (*n*=)	92	59	32
Anticoagulants	35 (38%)	22 (37%)	13 (41%)
Diabetes mellitus	9 (10%)	7 (12%)	2 (6%)
Hypertension	48 (52%)	31 (53%)	17 (53%)
Cardiovascular disease	40 (43%)	23 (39%)	18 (56%)
Hyper‐/Hypothyroidism	32 (35%)	19 (32%)	13 (41%)
Rheumatic disease	1 (1%)	0	1 (3%)
Infectious disease	0	0	0
Current smoker	21 (23%)	19 (32%)	1 (3%)
Former smoker	13 (14%)	13 (22%)	0
Never smoker	58 (63%)	27 (46%)	31 (97%)
Alcohol consumption	24 (26%)	21 (36%)	2 (6%)

Abbreviations: AR, antiresorptive therapy; IR, irradiation.

In the IR group, 46 patients (78%) had an oral squamous cell carcinoma as primary disease; four patients (7%) were treated for adenocarcinomas and four patients for carcinoma of unknown primary (CUP). Regarding tumor staging, four patients were classified as stage *T*_1_, 15 as stage *T*_2_, 12 as stage *T*_3_ and 21 as stage *T*_4_. In seven patients, the original tumor stage could not be ascertained due to missing records.

In 16 patients (27%), no surgical reconstruction of soft or hard tissues was necessary. Among the patients who underwent microsurgical reconstruction, radial forearm flaps (31%), fibula flaps (25%) and iliac crest flaps (15%) were used. The mean interval between transplantation and implant placement was 23 months (SD 22 months, median 14 months, range 3–100 months). Vestibuloplasty or free gingival grafting was performed in 26 patients (44%) after the closed healing period and prior to abutment connection, but never simultaneously.

The mean cumulative radiation dose was 63 Gy (SD 6 Gy, min. 45 Gy, max. 74 Gy). In 14 patients, the cumulative radiation dose was not available.

The mean interval from the end of radiotherapy to implant placement was 67 months (SD 63 months, range 6–276 months). Osteonecrosis had been previously diagnosed in 22 of the 59 patients (37%) and preventive dental extractions had been performed in 42 patients (71%).

The AR group comprised 32 patients (29 females, 3 males) with a mean age of 72 years (SD: 9; range: 55–87). Indications for bisphosphonate therapy were osteoporosis (*n* = 25) or malignancies (*n* = 7). Among the seven patients with malignant disease, metastases were already present. These patients received adjunct therapies, including antihormonal therapy (*n* = 3), chemotherapy (*n* = 1) or a combination of both (*n* = 3). The mean duration of AR therapy was 55 months (SD: 44; range: 4–192).

In two (6%) of the 32 patients, implants were placed in the neomandible or neomaxilla, meaning after microvascular anastomosed tissue transfer. Among these 10 implants, no implant loss was observed during the observation period.

### 3.2 Implant Level

A total of 92 patients received 369 implants, with a mean of four implants per patient (SD 2.2; range 1–10 implants). Overall, 46% of implants were placed in the interforaminal region of the mandible (positions 034–044). Sutures were removed after an average of 12 days (SD 13) and second‐stage surgery was conducted after 4 months (SD 2). Vestibuloplasty was necessary in 35 of the 92 patients (38% overall; IR: 44%, AR: 25%).

In the IR group 265 implants were inserted. Patients received a mean of 4.5 implants (SD 2.1, range 1–10 implants). At the end of the observation period, implant survival was 92% (244 implants). Implant failure occurred in 10 of 59 patients (17%) at the patient level. Implant loss occurred after a mean of 15 months (SD 14, range 3–44 months).

Ninety‐seven implants were placed in the AR group, with patients receiving a mean of three implants (SD 1.9; range 1–8 implants). At the end of the observation period, 95 implants were still in function, yielding an implant survival rate of 98%. Implant loss was limited to one patient, who lost two implants after 28 months. Thirty‐six percent of the implants were placed in the mandibular interforaminal region.

Suture removal was performed after a mean period of 11 days (SD 4).

### 3.3 Primary Endpoint

**FIGURE 1:**
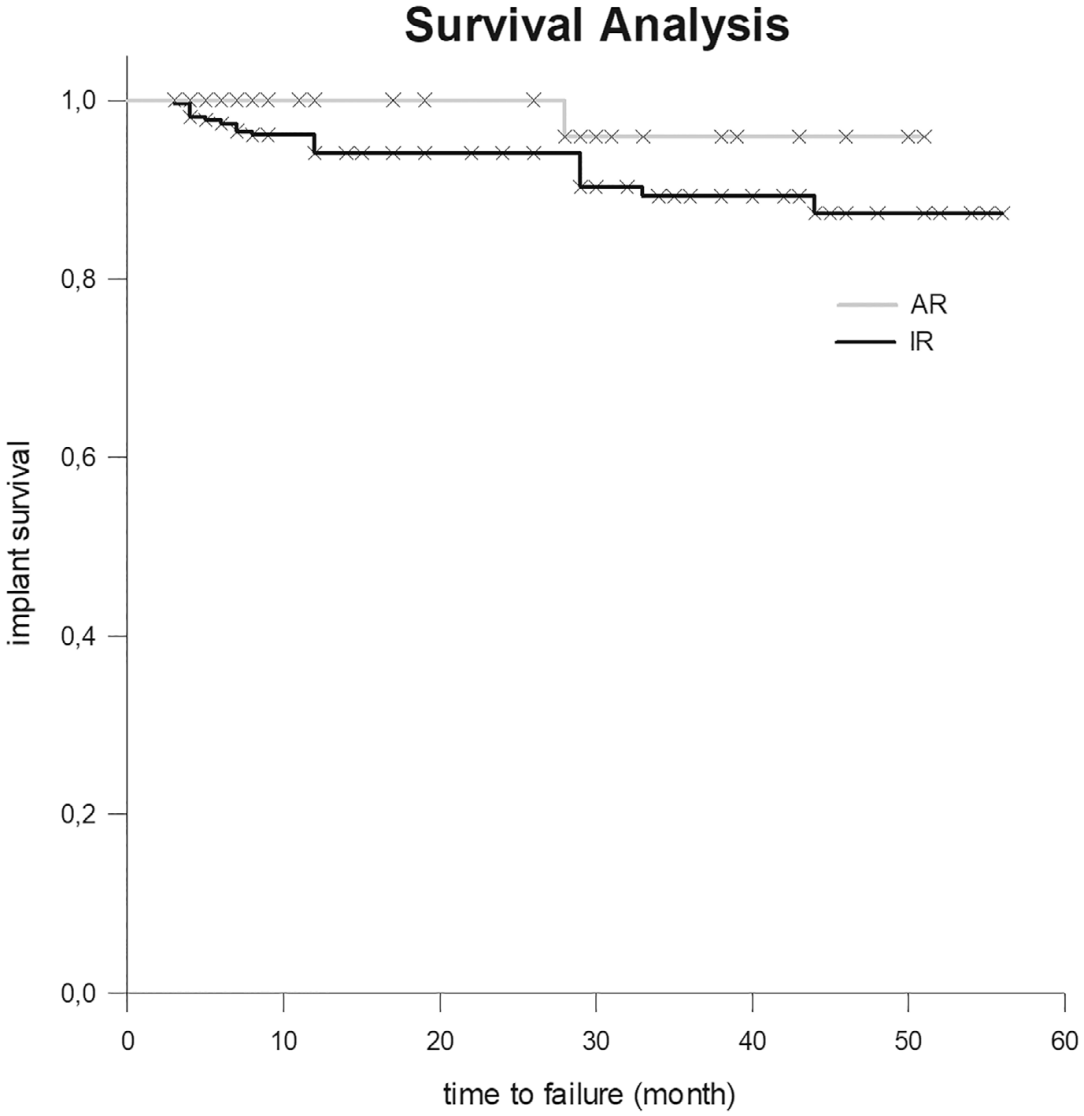
Kaplan–Meier diagram of implant survival for both groups: Irradiation (IR) and antiresorptive therapy (AR).

During a mean observation period of 25 months (SD 16 months), implant loss occurred in 11 out of 92 patients (IR: 10, AR: 1), corresponding to a patient level loss of 11.96%. At the implant level, 23 out of 369 implants were lost, resulting in an implant survival rate of 94%. On average, implant loss occurred after a mean period of 16 months (SD 13 months, range 3–44 months; Figure 1).

The predictors of implant failure at the implant level were identical in the total cohort and in the clinical subgroup including: neomandible implantation, small implant diameter (< 4.2 mm), diabetes mellitus, active smoking, and placement of the implant in the maxilla. In the IR group, a time interval of less than 60 months between radiotherapy and implantation was additionally associated with increased failure risk (Table 1).

### 3.4 Secondary Endpoints

Radiographs suitable for evaluation for peri‐implant marginal bone level and bone loss were available at the following time points: *T*_0_ for 74 patients, *T*_1_ for 51 patients, *T*_2_ for 18 patients, and *T*_max_ for 44 patients.

The time points of radiographic diagnostics averaged as follows: *T*_1_: 6 months (SD 1 month), *T*_2_: 12 months (SD 2 months) and *T*_max_: 34 months (SD 10 months).

Statistical analysis using the Mann–Whitney *U* test revealed significant differences (*p* < 0.001) in the IR risk group when comparing the values at *T*_0_ with those at *T*_1_, *T*_2_ and *T*_max_. The comparison between *T*_1_ and *T*_2_ also showed a significant difference (*p* < 0.001), while the comparison between *T*_2_ and *T*_max_ revealed no significant difference (*p* = 0.411).

For the AR therapy risk group, the analysis showed significant differences (*p* < 0.001) when comparing the values at *T*_0_ with those at *T*_1_, *T*_2_ and *T*_max_. The comparison between *T*_1_ and *T*_2_ showed no significant difference (*p* = 0.992), whereas the comparison between *T*_2_ and *T*_max_ demonstrated a significant difference (*p* = 0.043).

The comparison of the IR and AR risk groups using the Mann–Whitney *U*‐test revealed significant differences for the values at *T*_0_ (*p* < 0.001), *T*_1_ (*p* = 0.004), *T*_2_ (*p* < 0.001) and *T*_max_ (*p* < 0.001).

**FIGURE 2:**
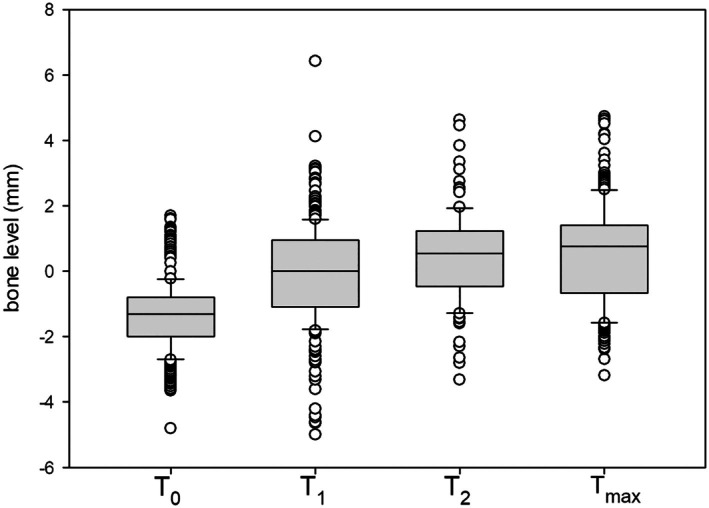
Box‐whiskers plots of marginal bone levels, analysis of the risk group irradiation (IR).

**FIGURE 3:**
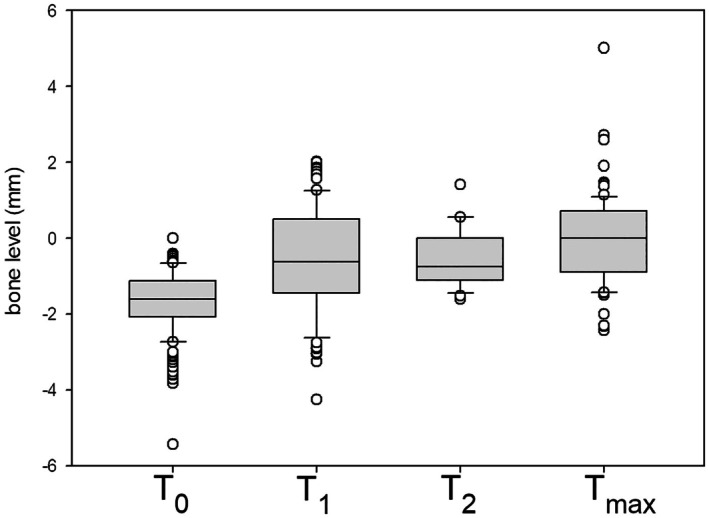
Box‐whisker plots of marginal bone levels, analysis of the risk group antiresorptive therapy (AR).

For all implants, the differences between the mesial and distal measurement values at *T*_1_, *T*_2_, and *T*_max_ and the respective values at *T*_0_ were calculated to determine bone resorption over time (Tables 4 and 5, Figures 2 and 3).

**TABLE 4:** Results for marginal bone loss in the irradiation study group (IR).

	*T*_0_	*T*_1_	*T*_2_	*T*_max_
Sample mean	−1.34	−0.10	0.47	0.57
Median	−1.31	0.00	0.56	0.76
SD	1.01	1.52	1.41	1.51
Max. values	−4.80	−5.00	−3.31	−3.18
Min. values	1.70	6.43	4.64	4.74

**TABLE 5:** Results for marginal bone loss in the antiresorptive therapy group (AR).

	*T*_0_	*T*_1_	*T*_2_	*T*_max_
Sample mean	−1.69	−0.64	−0.53	−0.09
Median	−1.62	−0.63	−0.76	0.00
SD	0.86	1.40	0.78	1.17
Max. values	−5.43	−4.26	−1.62	−2.44
Min. values	0.00	2.01	1.41	5.01

In the IR risk group, the Mann–Whitney *U* test comparing the values at *T*_1_ with those at *T*_2_ and *T*_max_ showed significant differences (*p* < 0.001). No significant differences were observed when comparing the values at *T*_2_ and *T*_max_ (*p* = 0.4).

In the AR risk group, comparison between *T*_1_ and *T*_2_ revealed no significant difference (*p* = 0.72). The comparison of *T*_1_ and *T*_max_ yielded a significant difference (*p* = 0.035), while the comparison of *T*_2_ and *T*_max_ showed no significant difference (*p* = 0.1).

When comparing the IR and AR risk groups using the Mann–Whitney *U* test, there were no significant differences at *T*_1_ (*p* = 0.661) and *T*_max_ (*p* = 0.051). However, a significant difference was observed at *T*_2_ (*p* = 0.007).

At the implant level, the LASSO‐regularized logistic regression model identified several variables as significantly associated with an increased risk of implant failure (Table 1).

**FIGURE 4:**
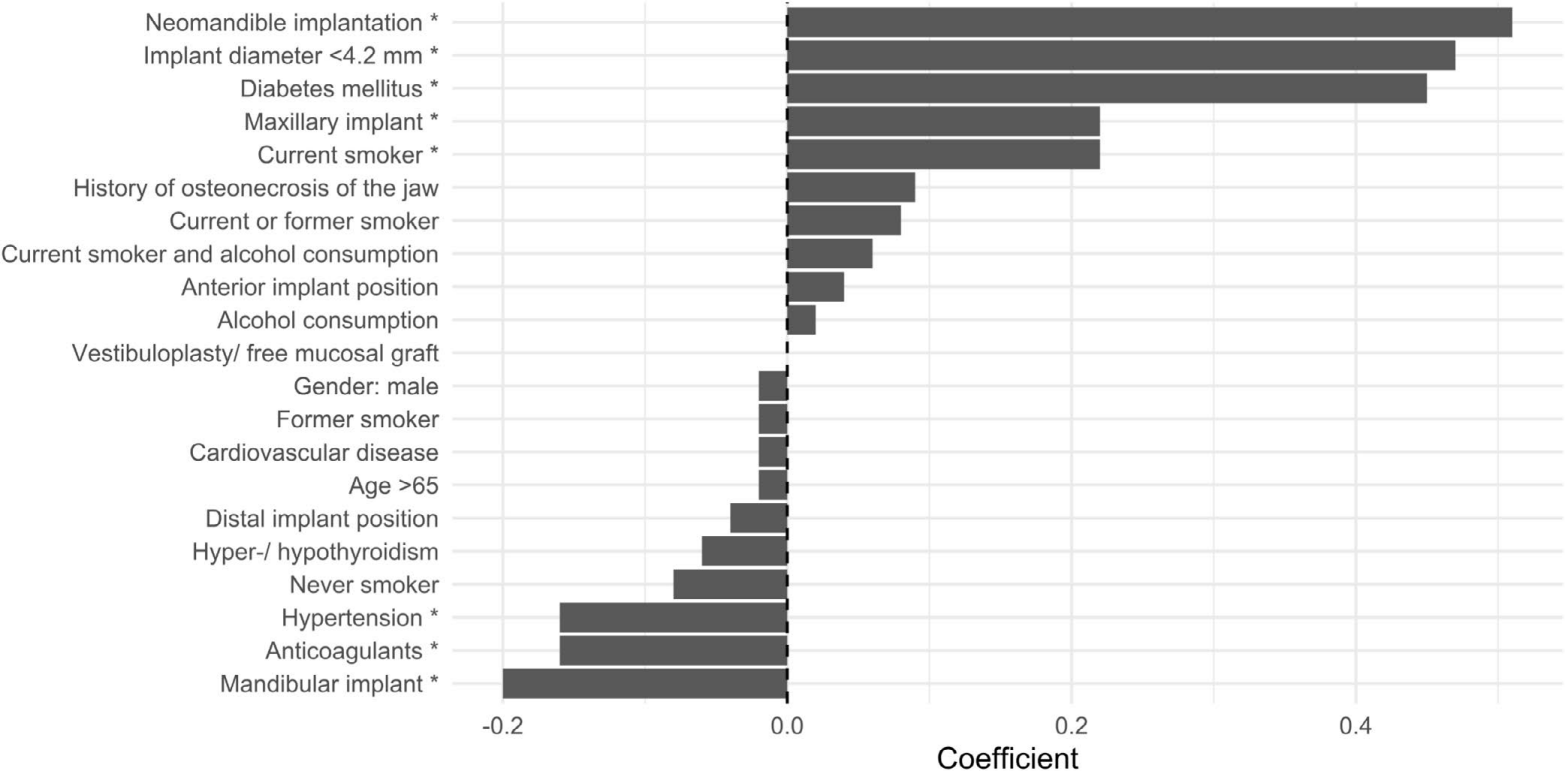
Coefficients of the total cohort (dependent variable: Implant failure).

**FIGURE 5:**
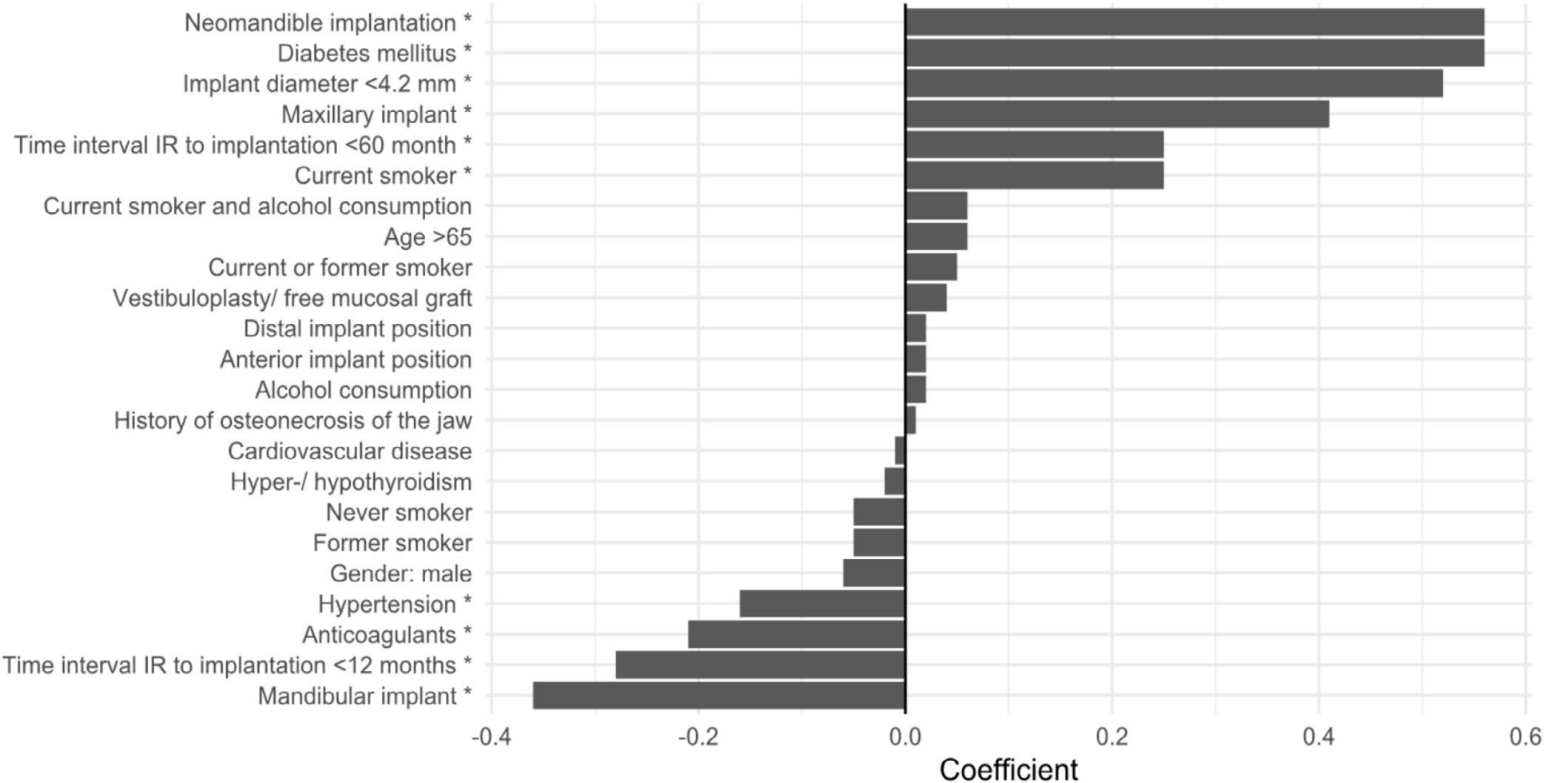
Coefficients of the irradiation risk group (IR) (dependent variable: Implant failure).

In both the total cohort and the IR subgroup, the most prominent predictors were implantation in the neomandible, smaller implant diameter (< 4.2 mm) and diabetes mellitus. These variables consistently demonstrated high and statistically significant coefficients, indicating a robust association with elevated failure risk. Furthermore, active smoking and implant placement in the maxilla were also significantly associated with a higher likelihood of failure (Figures 4 and 5).

Other variables, including age > 65 years, gender, cardiovascular disease, thyroid dysfunction, alcohol consumption, and smoking history (former or never smokers), were not significantly associated with implant failure. Conversely, anticoagulant use and hypertensive disease exhibited statistically significant negative coefficients.

In the IR patient cohort additionally, a time interval of less than 60 months between radiotherapy and implantation was associated with increased risk of failure. An interval of less than 12 months was not linked to any further increase in risk. This is primarily caused by the absence of implant failure cases in the analyzed cohort and should therefore be interpreted with caution.

Clinical evaluation of biological complications was conducted at follow‐up visits, the data of which are summarized in Table 6.

**TABLE 6:** Results of secondary endpoints in both groups: Irradiation (IR) and antiresorptive therapy (AR) at three different time points. Dehiscence, infection, bleeding on probing, probing depth.

	IR			AR		
	*T*_1_ (8 weeks)	*T*_2_ (6 months)	*T*_3_ (12 months)	*T*_1_ (8 weeks)	*T*_2_ (6 months)	*T*_3_ (12 months)
Dehiscence						
No	59	52	45	32	29	21
Yes	0	5	2	0	0	0
Infection						
No	59	56	47	32	29	20
yes	0	1	0	0	0	1
BOP						
No		50	41		28	20
Yes		7	6		1	1
Probing depth						
< 3.5 mm		50	43		29	21
> 3.5 mm		7	4		0	0

## 4 Discussion

Dental rehabilitation in patients with a risk profile for developing ONJ presents a significant challenge for the practitioner. Patients undergoing or having undergone AR/IR therapy in the head and neck region are considered high‐risk patients for dental implants. Implant placement in these cases is recommended only after thorough risk assessment, strict adherence to specific surgical protocols, and compliance with preventive measures (Groetz et al. 2016; Ristow et al. 2021; Schiegnitz et al. 2018).

The aim of such assessment is to identify cases where implant‐supported rehabilitation will significantly improve swallowing, speech, and mastication, thus enhancing quality of life while minimizing the risk of ONJ. This study aimed to investigate whether, with standardized surgical management, the risk of complications in these high‐risk patient groups remains reasonable, achieving predictable long‐term treatment success.

A total of 92 patients with 369 implants were included in this retrospective single‐center analysis, comprising 59 patients in the IR group and 32 in the AR group, with one patient in both groups. In the IR group, 244 out of 265 implants (92%) remained functional, with implant failure occurring in 10 patients (17%) at a mean of 15 months post‐placement. In the AR group, 97 implants were placed, with 95 (98%) remaining functional. Overall, implant failure occurred in one patient who lost two implants after 28 months.

The survival rates, based on a benefit–risk assessment, were acceptable in both groups. However, the strength of the indication and a consistent risk assessment for developing ONJ were strictly weighed The risk of AR‐ONJ is significantly influenced by factors such as type, dose, and duration of AR medication (indirectly by the underlying disease) as well as concomitant immune‐modulating conditions and medications (Ristow, Ruckschloss, et al. 2023; Ristow, Schnug, et al. 2023). The risk of IR‐ONJ, on the other hand, is primarily determined by the irradiation field, irradiation procedure, and total dose, along with resulting complications such as xerostomia and tissue fibrosis (Ristow, Birgel, et al. 2023). For both patient groups, oral hygiene plays a crucial role, directly impacting surgical success and indirectly influencing implant survival and the risk of developing ONJ over time (Seki et al. 2024).

In this study, diabetes (*β* = 0.45; *p* < 0.01) and current smoking (*β* = 0.22; *p* = 0.02) were the two comorbidities associated with an increased risk of implant loss.

All patients underwent surgery in strict adherence to a modified protocol based on German guidelines, incorporating all preventive measures (Groetz et al. 2016; Ristow et al. 2021; Schiegnitz et al. 2018). These included prolonged perioperative antibiotic prophylaxis, bone‐sparing techniques, primary plastic wound closure, and submerged implant healing to minimize bone infections, consistent with the infection‐dependent pathogenesis theory (Troeltzsch et al. 2023). Furthermore, immediate implant loading and bone augmentation procedures were avoided to further reduce ONJ risk.

Among the trigger factors for ONJ described in the literature, prosthesis pressure points are frequently found and are thus classified as a risk factor. Niibe et al. (2015) retrospectively evaluated 128 patients receiving intravenous bisphosphonates for unspecified reasons, categorizing them into three groups. The first group of 34 patients had removable dentures, of whom 11 patients developed ONJ (32%). In the second group, also consisting of 34 patients with fixed partial dentures, only five cases of ONJ were observed (15%). The third group, comprising 60 patients without dentures, experienced the lowest incidence, with five cases of ONJ (8%).

Similarly, Hasegawa et al. (2012) reported that patients with prostheses had a shorter average time to ONJ onset compared to those without prostheses. A recent study of 6018 women with stage I–III breast cancer further highlighted this association, reporting 48 cases of ONJ. Among patients with full dentures, the incidence was 2.7%, significantly higher than the 0.8% observed in those with partial or no removable dentures.

Implants offer a prosthetic advantage by reducing mucosal load and eliminating pressure points associated with conventional removable dentures. This not only improves the prognosis of the residual dentition but may also indirectly reduce the risk of ONJ. Consequently, the indication for implant placement in these patients should always be carefully evaluated.

In this study, patients receiving simultaneous bone augmentation were deliberately excluded, as such procedures demand strict indication assessments. Bone augmentation poses significant challenges for the recipient tissue, particularly in terms of vascularization, resorption, and new bone formation, processes often compromised in patients undergoing or post AR/IR therapy. Consequently, augmentation indirectly increases the risks of implant failure and ONJ. Few studies have explored this topic.

Khoury and Hidajat (2016) examined 15 osteoporosis patients, nine treated with oral bisphosphonates and six with intravenous bisphosphonates (alendronate, clodronate, ibandronate, risedronate), who underwent lateral and/or vertical augmentations in 47 regions and 14 sinus lifts. A total of 71 implants were placed, with only one immediately loaded implant lost after 5 months. No cases of ONJ were reported. Although data on bone augmentation in patients receiving AR therapy remain limited, such procedures must undergo stringent indication testing to avoid unnecessarily increasing the risk of ONJ (Groetz et al. 2016; Schiegnitz et al. 2018).

In the present study, the use of reduced‐diameter implants (< 4.2 mm) in sites with limited bone volume was associated with a higher likelihood of implant failure (*β* = 0.47; *p* < 0.01).

In line with previous studies, a significantly lower implant survival rate in IR patients compared to non‐IR patients has been reported (Hoffmann et al. 2024; Schiegnitz et al. 2014, 2022; Shokouhi and Cerajewska 2022). In the study by Hoffmann et al. (2024), implant survival rates of 96.2% in non‐IR patients and 87.6% in post‐IR patients were observed (*p* = 0.034). In an umbrella review involving 5487 head and neck tumor patients and nearly 25,000 implants placed, Zarzar et al. (2024) calculated an implant survival rate of 86.2% in IR patients and 95.2% in non‐IR patients.

In a study by Lombardo et al. (2023) implant survival after a five‐year observation period was 94.2% in patients without IR and 83.5% in those who had undergone IR when using free fibula grafts (Lombardo et al. 2023). Similarly, Pellegrino et al. (2018) reported a five‐year implant survival rate of 83.5% in IR patients and 94.2% in non‐IR patients with microvascular fibular grafts. Kaiser et al. (2024) observed an implant survival rate of 86.3% over a 48.7‐month observation period for 227 implants placed in microvascular fibular, iliac crest, or scapular grafts.

Ettl et al. (2020) identified implantation into transplanted bone in patients with a history of IR as a risk factor for implant failure, with an odds ratio of 2.4. This finding was corroborated by a meta‐analysis by Schiegnitz et al. (2014) which emphasized that implant placement in native bone should be prioritized, as implant survival is significantly compromised in cases of bone augmentation combined with IR (Dholam and Gurav 2012; Salinas et al. 2010).

A more recent systematic review by Schiegnitz et al. (2022) further demonstrated that, despite an overall implant survival rate of 87.8%, survival in irradiated bone remained significantly lower than in non‐irradiated bone (OR = 1.97). Moreover, irradiated non‐native bone was more frequently associated with implant loss compared to irradiated native bone (OR = 2.26).

In the present study, a statistically significant association was also found between implant failure and implantation in the neomandible (*β* = 0.51; *p* < 0.01).

The optimal timing for implant placement remains a topic of debate. In IR patients, Sriram et al. reported in a meta‐analysis that implant survival was significantly lower in those who received more than 60 Gy of radiation (82.8%) compared to those receiving less than 60 Gy (90.1%). Implant placement more than 1 year after completing IR therapy showed a marked improvement in survival rates (96.7% vs. 82.5%; Sriram et al. 2024). For patients in the AR group, bone regeneration may be impaired due to AR medication, with the extent of impairment dependent on the dose, duration, and type of medication. This can prolong the osseointegration phase, suggesting that extended healing times may be beneficial. Additionally, clinical and radiological indicators of compromised healing, such as sharp bone edges or persistent alveoli, which may reflect impaired soft tissue healing, bone remodeling, or new bone formation, should be carefully assessed and factored into the decision‐making process before implantation.

In the current study, the cumulative radiation dose could be determined for 45 of the 59 patients, resulting in a mean dose of 63 Gy (SD 6 Gy, median 66 Gy, min. 45 Gy, max. 74 Gy). The mean interval between the end of radiotherapy and implantation was 67 months (±SD 63 months, median 37 months, min. 6 months, max. 276 months). Implantation was always performed at least 6 months after the end of IR. Among the six patients who received their implants 6–12 months post‐IR, no implant failure was observed. At the implant level, a significant association was noted between implantation less than 60 months after IR and implant loss (*ß* = 0.25; *p* = 0.02). Various authors recommend waiting 6–12 months after completion of IR before implant placement to avoid increased rates of implant failure (Anderson et al. 2013; Claudy et al. 2015; Toneatti et al. 2021). Current scientific consensus suggests that after IR, there may be a transient period of improved healing and increased cell turnover, whereas the negative effects of hypovascularization become more pronounced over time (Toneatti et al. 2021; Visch et al. 2002). Numerous literature sources indicate that in IR patients, implant losses frequently occur shortly after insertion and are more common in the maxilla. This may be explained by the higher bone density and better primary stability in the mandible (Buddula et al. 2012; Kovacs 2000; Sammartino et al. 2011). Kaiser et al. (2024) reported an implant loss rate of 23.8% in the maxilla and 11.4% in the mandible. A similar tendency appears in the healthy general population; for instance, Fouda calculated an implant loss rate of 1.96% in the mandible and 3.14% in the maxilla (Fouda 2020). In the present study, implantation in the maxilla showed a significant association with implant loss at the implant level (*β* = 0.22; *p* < 0.01 in the total cohort and *β* = 0.41; *p* < 0.01 in the IR subgroup).

Sufficient and stable peri‐implant soft tissue is crucial for long‐term inflammation‐free outcomes. Hoffmann et al. noted that 67% of the investigated tumor patients required peri‐implant soft tissue surgery, highlighting the importance of stable tissue management (Hoffmann et al. 2024; Longoni et al. 2019). Patient motivation and ability to maintain oral and peri‐implant hygiene are essential to minimizing inflammatory complications and should be integral to the decision‐making process given the potential severity of inflammatory implant complications.

The differing implant outcomes between the IR and AR groups can be explained by several physiological and clinical mechanisms:

IR in the head and neck region causes vascular and cellular damage, leading to a reduced microvascular density. This diminished blood supply impairs the delivery of oxygen and nutrients to the bone tissue, resulting in limited cellular proliferation and delayed wound healing. IR also induces increased fibrosis in the affected tissue. Fibrotic tissue exhibits reduced elasticity and a diminished capacity for regeneration, which in turn hinders the osseointegration of implants. Due to impaired wound healing and decreased vascular supply, the risk for osteoradionecrosis is elevated. This pathophysiological condition can lead to a higher implant failure rate, as the irradiated bone regions are less capable of regeneration.

AR medications such as bisphosphonates or denosumab primarily inhibit osteoclast activity, leading to reduced bone resorption and often an increase in bone mineral density. Although this alters bone metabolism, the local vascular supply generally remains intact. Unlike IR, AR medications do not cause comparable levels of vascular damage or fibrosis. Consequently, the capacity for bone regeneration and osseointegration is largely maintained, provided that preventive measures are observed. Furthermore, the systemic effects of AR therapy tend to be more uniformly distributed than the focal damage induced by IR.

In both patient groups, strict preventive measures and standardized surgical protocols are paramount. However, in IR patients, the bone substrate is intrinsically less regenerative due to radiation‐induced damage. In contrast, when procedures in AR patients are performed in areas of healthy bone, osseointegration is less compromised.

The higher implant survival rate observed in AR patients compared to IR patients is primarily attributable to less severe local tissue damage. IR results in permanent changes, such as reduced vascularity, fibrosis, and diminished cellular regenerative capacity, which impair osseointegration and wound healing. Although the bone in AR patients is systemically affected, the local vascular supply and regenerative tissue structure remain largely preserved, allowing stable implant integration when proper indications are established and preventive measures are strictly followed. These physiological differences explain the differing clinical outcomes observed between the two patient groups.

The retrospective approach is a limitation of this study. However, all patients were planned, monitored, and documented by the same experienced specialists in our dedicated consultation hour for IR or AR‐treated patients, following a predefined protocol to ensure data consistency. Another limitation is the heterogeneity of the patient population. Although strict inclusion and exclusion criteria were applied to minimize confounding factors as much as possible, it is important to acknowledge that factors such as smoking and alcohol consumption, which are commonly present in head and neck cancer patients, remain difficult to completely exclude. Smoking, in particular, is a well‐known risk factor associated with impaired healing and an increased incidence of peri‐implant diseases (Herrera et al. 2023).

Despite these risks, these patients were carefully counseled about the higher risk of complications, and treatment was only pursued after a thorough risk–benefit assessment and obtaining informed consent.

## 5 Conclusion

After thorough risk assessment and evaluation of the implant indication, implants in medically compromised patients undergoing or having received AR or IR can be a viable option if preventive measures are strictly followed. Implants placed after AR exhibit higher survival rates and less marginal bone loss compared to those placed after IR.

## Author Contributions

C.M.: conceptualization, methodology, supervision, writing – original draft. F.S.: software, data curation, investigation, formal analysis, visualization, writing – review and editing. M.S.: validation, writing – review and editing. T.R.: validation, writing – review and editing. G.S.: validation, writing – review and editing. J.H.: methodology, supervision, resources, writing – review and editing. O.R.: conceptualization, methodology, formal analysis, supervision, project administration, writing – original draft. All authors agree to be accountable for all aspects of the work, they will ensure that questions related to the accuracy or integrity of any parts of the work are appropriately investigated and resolved.

## Ethics Statement

The study protocol was approved by the Medical Faculty's Ethics Committee (approval no. S‐373/2022). All patients provided informed consent for inclusion in the study.

## Conflicts of Interest

The authors declare no conflicts of interest.

## Data Availability

The data supporting the findings of this study are available from the corresponding author upon reasonable request.
